# Reproducibility of Kidney Perfusion Measurements With Arterial Spin Labeling at 1.5 Tesla MRI Combined With Semiautomatic Segmentation for Differential Cortical and Medullary Assessment

**DOI:** 10.1097/MD.0000000000003083

**Published:** 2016-03-18

**Authors:** Matthias Hammon, Rolf Janka, Christian Siegl, Hannes Seuss, Roberto Grosso, Petros Martirosian, Roland E. Schmieder, Michael Uder, Iris Kistner

**Affiliations:** From the Department of Radiology (MH, RJ, HS, MU), University Hospital Erlangen, Friedrich-Alexander-Universität Erlangen-Nürnberg (FAU), Maximiliansplatz, Erlangen, Germany; Department of Computer Graphics (CS, RG), Friedrich-Alexander-Universität Erlangen-Nürnberg, Cauerstraße, Erlangen, Germany; Experimental Radiology, Department of Diagnostic and Interventional Radiology (PM), University Hospital Tübingen, Otfried-Müller-Straße, Tübingen, Germany; and Department of Nephrology and Hypertension (RES, IK), University Hospital Erlangen, Ulmenweg, Erlangen, Germany.

## Abstract

Magnetic resonance imaging with arterial spin labeling (ASL) is a noninvasive approach to measure organ perfusion. The purpose of this study was to evaluate the reproducibility of ASL kidney perfusion measurements with semiautomatic segmentation, which allows separate quantification of cortical and medullary perfusion.

The right kidneys of 14 healthy volunteers were examined 6 times on 2 occasions (3 times at each occasion). There was a 10-minute pause between each examination and a 14-day interval between the 2 occasions. Cortical, medullary, and whole kidney parenchymal perfusion was determined with customized semiautomatic segmentation software. Coefficient of variances (CVs) and intraclass correlations (ICCs) were calculated.

Mean whole, cortical, and medullary kidney perfusion was 307.26 ± 25.65, 337.10 ± 34.83, and 279.61 ± 26.73 mL/min/100 g, respectively. On session 1, mean perfusion for the whole kidney, cortex, and medulla was 307.08 ± 26.91, 336.79 ± 36.54, and 279.60 ± 27.81 mL/min/100 g, respectively, and on session 2, 307.45 ± 24.65, 337.41 ± 33.48, and 279.61 ± 25.94 mL/min/100 g, respectively (*P* > 0.05; *R*^2^ = 0.60/0.59/0.54). For whole, cortical, and medullary kidney perfusion, the total ICC/CV were 0.97/3.43 ± 0.86%, 0.97/4.19 ± 1.33%, and 0.96/4.12 ± 1.36%, respectively. Measurements did not differ significantly and showed a very good correlation (*P* > 0.05; *R*^2^ = 0.75/0.76/0.65).

ASL kidney measurements combined with operator-independent semiautomatic segmentation revealed high correlation and low variance of cortical, medullary, and whole kidney perfusion.

## INTRODUCTION

In diverse clinical situations, it is of interest to evaluate renal function (estimation of the glomerular filtration rate and renal tissue oxygenation). In this context, it is of importance to assess renal perfusion.^1,2^ Impairment of renal perfusion is a marker of organ damage in a variety of morbidities, including hypertension, obesity, metabolic syndrome, diabetes, and atherosclerosis.

There are different methods available for kidney perfusion measurements. In diverse clinical settings, renal function is usually assessed with the estimated glomerular filtration rate, which is commonly estimated from serum values of endogenous markers (e.g., serum creatinine).^3,4^ However, these surrogate parameters have a limited sensitivity to alterations in renal physiology. Furthermore, changes in these parameters may occur at a later stage during the development of kidney disease or may even be normal despite a significant compromise in renal perfusion (e.g., in the presence of renal artery stenosis). These limitations are not only important for patient care but also in clinical research.

Clearance techniques are conventionally used to measure effective renal blood flow, with the para-aminohippurate (PAH) clearance being the gold standard.^5^ However, this process is labor-intensive, time consuming, expensive, invasive, and bears potential side effects, such as anaphylaxis. Furthermore, it does not allow assessment of the different compartments of the kidney. Doppler sonography is a noninvasive procedure to detect renal artery stenosis or alterations in the peripheral vessel resistance, but does not allow accurate quantification of the tissue perfusion^6–8^ and does not reflect renal vascular resistance.^9^ Moreover, measurements of global kidney function are not suitable for the detection of diminished function in restricted areas or compartments (e.g., cortex, medulla).^10^ Radionuclide scintigraphy is invasive due to the use of an exogenous radioactive tracer, limiting its use in clinical trials and under special conditions (e.g., pregnancy).^11,12^ Dynamic perfusion studies performed using both computed tomography (CT) or magnetic resonance imaging (MRI) require administration of an exogenous contrast compound that may be nephrotoxic (e.g., in the case of iodinated contrast used during CT examinations) and that also involves an ionizing radiation exposure. Paramagnetic gadolinium-based contrast agents for MRI, while generally safe, are not suitable for use in patients with advanced renal impairment due to the minor but potential risk for the development of nephrogenic systemic fibrosis.^13^

Therefore, a noninvasive, reproducible, and quick method to quantify kidney perfusion is desirable. Due to its high perfusion rate, the kidney is an appropriate candidate for perfusion imaging with MRI utilizing arterial spin labeling (ASL), which uses magnetically labeled protons in the blood as an endogenous contrast agent. Hence, the administration of a contrast agent is not necessary. In recent years, several ASL techniques, including the flow-sensitive alternating inversion recovery (FAIR) technique, have been developed mainly to measure perfusion of the brain.^14,15^ With respect to the kidneys, ASL approaches, including the FAIR True-FISP technique, have recently been demonstrated to produce clinically valuable perfusion measurements.^16–19^ Several studies have investigated changes in renal perfusion with MRI techniques.^16,20,21^ A significant decrease in renal perfusion was reported in patients with renal artery stenosis.^17,22^ Regional differences of microcirculation have been reported in renal cell carcinomas^23,24^ and renal allografts.^25^ Most ASL imaging in the literature was carried out with widely available MR scanners having a field strength of 1.5 Tesla.^17,26–28^ Therefore, ASL represents a noninvasive method to measure renal perfusion without exposure to ionizing radiation or exogenous contrast agents.

One current constraint is the time consumption and operator dependence required for image analysis. Gillis et al^29^ reported a mean image analysis time of approximately 30 minutes per kidney. Therefore, the purpose of this study was to investigate the reproducibility of ASL combined with semiautomatic segmentation of the renal compartments, which allowed the differential quantification of cortical and medullary perfusion at 1.5 Tesla MRI.

## METHODS

### Patient Characteristics and Study Design

This pilot study included 14 subjects; 5 healthy volunteers and 9 hypertensive patients with controlled blood pressure. Mean age was 48 ± 13 years and 9 subjects of the total cohort were male (5 female). The body mass index of all subjects was on average 27 ± 5.2 kg/m^2^. All subjects had an estimated glomerular filtration rate >60 mL/min/1.73 m^2^ (MDRD formula) at baseline. The average office blood pressure at baseline was 133 ± 16/82 ± 10 mm Hg. The study recruitment, which was started in July 2014, was conducted by advertisement in local newspapers. All patients gave written informed consent and the study was executed according to the Declaration of Helsinki and “good clinical practice” (GCP) guidelines. There were 2 drop-outs, one caused by an abnormal anatomy of the kidney and the other caused by breathing artifacts. Subjects were examined 2 times with an interval of 2 weeks. At each time point, 3 measurements of 10 minutes each with a rest period of 10 minutes between the measurements were performed. Overall, 6 measurements on each patient were performed. At both time points the examinations were performed within 1 hour at the same time of day.

### Arterial Spin Labeling

ASL was performed with a 1.5 Tesla MRI scanner (Magnetom Aera, Siemens, Erlangen, Germany) using a FAIR True-FISP sequence. The FAIR and true fast imaging with steady-state precession (True-FISP) approach combines a FAIR perfusion preparation and a True-FISP data acquisition strategy. The perfusion measurement is based on 2 data acquisitions, one with a global inversion prepulse, followed by one with a slice selective inversion (FAIR). The prepulses lead to a labeling of blood water spins. In contrast to the global inversion prepulse, the slice selective inversion prepulse only labels the blood water spins inside the kidney but not the inflowing blood water spins. Subtraction of both images therefore reflects the local perfusion. A 3rd image without the FAIR preparation pulse was measured to normalize the signal intensities on each patient. The technical and theoretical background of the sequence has been previously described in detail.^16^

**FIGURE 1:**
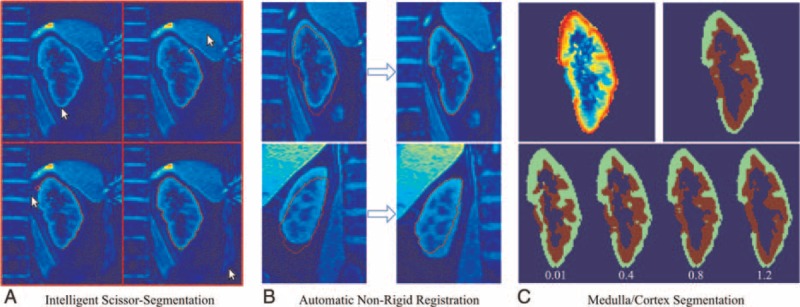
Processing of the images (from left to right). First, a semiautomatic segmentation using intelligent scissors is performed (A). The resulting contour is registered onto all images of 1 series using nonrigid registration (B). By evaluating the registration results we can distinguish a good (B, top) from a bad registration (B, bottom). Cortex/medulla segmentation was performed using a k-means clustering algorithm (C). This clustering is based on the averaged registered global inversion images (C, top left). To enable the user to modify the final medulla/cortex segmentation a parameter α is premultiplied to the gray values and directly influences the boundary between medulla and cortex result (c, bottom). In the assessed cases, the parameter α was set to 1.0 (c, top right). However, the adjustment of α may be beneficial in particular cases.

MRI, as performed in the current study, is illustrated in Figure 1. All patients were examined in the supine position with a body-phased array coil (Siemens, Erlangen, Germany) in combination with a spine coil (Siemens, Erlangen, Germany). The FAIR True-FISP parameters included the following: repetition time, 4.9 ms; echo time, 2.5 ms; effective inversion time, 1200 ms; flip angle, 70°; field of view, 360 mm; and in-plane resolution, 2.3 × 1.2 mm. All images were measured during expiration in breath hold. Breath-hold time was 18 seconds. Slices were positioned in an oblique coronal orientation to match the longitudinal axis of both kidneys. Slice thickness was 8 mm. Care was given to similarly position the slices in all subjects, and crucial attention was made to match the same slice position in both study time points within one subject.

### Image Analysis

Perfusion images are computed from a pair of global/slice inversion images and an M0 image.^15,17^ Since this computation is performed at pixel level, precise registration of these images is crucial. However, patient movement during acquisition and fluctuations in the breath-hold results in a shift of the kidney. To counteract these effects, we chose a simultaneous segmentation/registration approach based on the software presented by Siegl et al.^30^ One image has to be segmented using the well-known semiautomatic intelligent scissor method proposed by Mortensen and Barrett^31^ (Figure 1 left). The resulting segmentation is then registered onto the remaining images using a very stiff nonrigid registration. This registration is physically motivated and tries to mimic the kidney movement during acquisition (translation, rotation, scaling, and shearing). Therefore, the contour of the intelligent scissor segmentation step is deformed such that it best fits the contour of the target kidney. By analyzing the residual error of the resulting contours within 1 image series, we are able to assess the quality of the registration and sort out acquisitions that do not show adequate image quality. In Figure 1B on top, the registration result is good. In the bottom example, the contour cannot be registered and still cuts through the kidney. This results in a much larger residual error. The corresponding image pair will be sorted out. Please note that we intentionally chose such a very stiff registration. Registering kidneys with more deformation would be possible; however, if this becomes necessary the acquisition plane has shifted too much and the data can no longer be compared in a meaningful way.

Since we are especially interested in distinct cortex and medulla perfusion values, an additional segmentation step was required. For this purpose, we use the average over the registered global inversion images (Figure 1C, top left). In pretests, we identified the global inversion images to show the best separation of gray values between calyces, medulla, cortex, and background. To separate these regions, we use a k-means clustering method^32^ on the pixel gray values. For increased robustness, we extended this gray value based approach by adding the distance to the kidney's center of gravity into the computation. This leads to coherently segmented regions and finally to a more robust and correct registration (Figure 1C, top right). To enable the user to modify the final medulla/cortex segmentation a parameter α is premultiplied to the gray values and directly influences the boundary between medulla and cortex (Figure 1C, bottom). In the assessed cases, α was set to 1.0. However, the adjustment of this parameter may be beneficial in special cases.

Since we were especially interested in correct cortical perfusion values, an additional step was introduced to further improve their quality. A morphological filter is used to ensure that no medullar or background pixels remain in the region of the segmented cortical area. Although this may lead to a slightly under-segmented cortex, the segmentation is guaranteed to only contain pixels completely belonging to the cortex.

The final image analysis in our experiments was always based on 8 acquired global/slice inversion image pairs and 1 M0 image. These images were segmented and registered using the previously described algorithm. Bad image pairs can be sorted out immediately by the registration software. We evaluated different computation schemes and settled on computing a perfusion image for every acquired image pair. From the image pairs showing a good registration, we used the 4 pairs showing the highest perfusion values. Taking the average over these perfusion images showed the best reproducibility, while maintaining a good separation between patients with different kidney functions.

### Statistical Analysis

Statistical analysis was performed using SPSS software version 21 (IBM, Armonk, NY). Continuous variables were expressed as mean ± standard deviation. For paired samples, a *t*-test was applied for parametric data. All tests were performed 2-sided, and *P* < 0.05 was considered to be statistically significant. Comparisons of renal perfusions between occasions 1 and 2 were made using paired *t*-tests. Pearson correlation coefficients were determined to show correlation between MRI measurements. Bland–Altman plots were prepared of the mean perfusion values against the difference between the values, with the 95% limits of agreement calculated as the mean difference plus or minus 1.96 times the standard deviation of the difference. The coefficient of variance (CV) was also calculated, which represents the ratio of the standard deviation to the mean.

## RESULTS

Mean perfusion of the whole kidney was 307.26 ± 25.65 mL/min/100 g, mean cortical perfusion was 337.10 ± 34.83 mL/min/100 g, and mean medullary perfusion was 279.61 ± 26.73 mL/min/100 g. Mean perfusion on session 1 was 307.08 ± 26.91 mL/min/100 g for the whole kidney, 336.79 ± 36.54 mL/min/100 g for the cortex, and 279.60 ± 27.81 mL/min/100 g for the medulla. On session 2 the corresponding perfusion results were 307.45 ± 24.65, 337.41 ± 33.48, and 279.61 ± 25.94 mL/min/100 g (*P* = 0.89, *R* = 0.78, *R*^2^ = 0.60; *P* = 0.87, *R* = 0.78, *R*^2^ = 0.59; *P* = 0.99, *R* = 0.73, *R*^2^ = 0.54), respectively. The CVs were 3.24 ± 1.54%, 3.80 ± 1.70%, and 3.27 ± 1.82% for the whole kidney, cortical, and medullary perfusions on session 1 and 2.30 ± 0.95%, 3.02 ± 1.21%, and 3.87 ± 2.07% on session 2, respectively. The corresponding intraclass correlations (ICCs) were 0.93, 0.95, and 0.94 on session 1 and 0.97, 0.96, and 0.92 on session 2, respectively.

**FIGURE 2:**
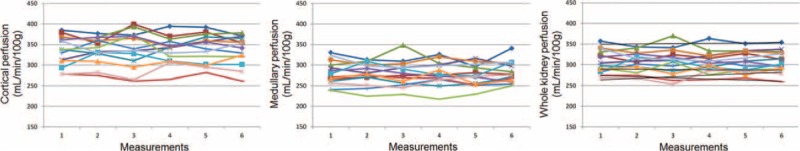
Results of arterial spin labeling kidney perfusion measurements of 14 healthy subjects at 1.5 Tesla. Kidney perfusion of each participant was measured 6 times (2 × 3 times with a 14-day interval). Participants are shown in different colors.

**FIGURE 3:**
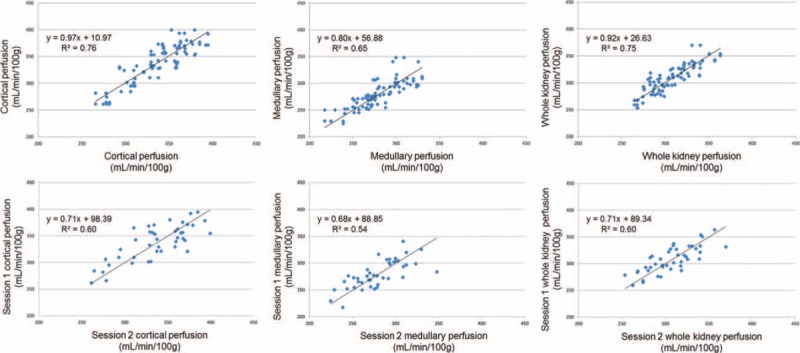
Correlation plots of all kidney perfusion measurements (upper) and of session 1 and 2 kidney perfusion measurements (lower) of 14 healthy subjects measured 6 times (2 × 3 times with a 14-day interval). *R*^2^ = coefficient of determination.

**FIGURE 4:**
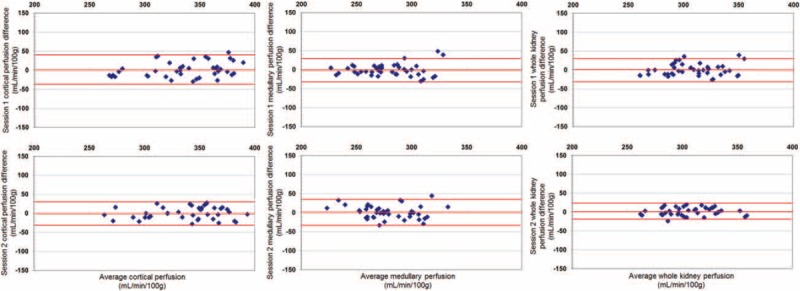
Bland–Altman plots showing the results of kidney perfusion measurements of session 1 and 2. Red lines show the means of the differences and the means of the differences ± 1.96 × the standard deviation of the differences.

The total ICC for cortical perfusion was 0.97, while the CV was 4.19% ± 1.33%. The ICC for medullary perfusion was 0.96, while the CV was 4.12% ± 1.36%. The ICC for the whole kidney perfusion was 0.97, while the CV was 3.43% ± 0.86%. Measurements did not differ significantly (*P* > 0.05) and showed a very good correlation (*R* = 0.86/0.87/0.81). The results of kidney perfusion measurements are shown in Figure 2 and Table 1 . Correlation is illustrated in Figure 3. Bland–Altman plots were prepared for the cortical, medullary, and whole kidney perfusion measurements at session 1 and 2 (Figure 4). These show good agreement between measurements, with a random distribution of means plotted against differences observed.

**TABLE 1:**
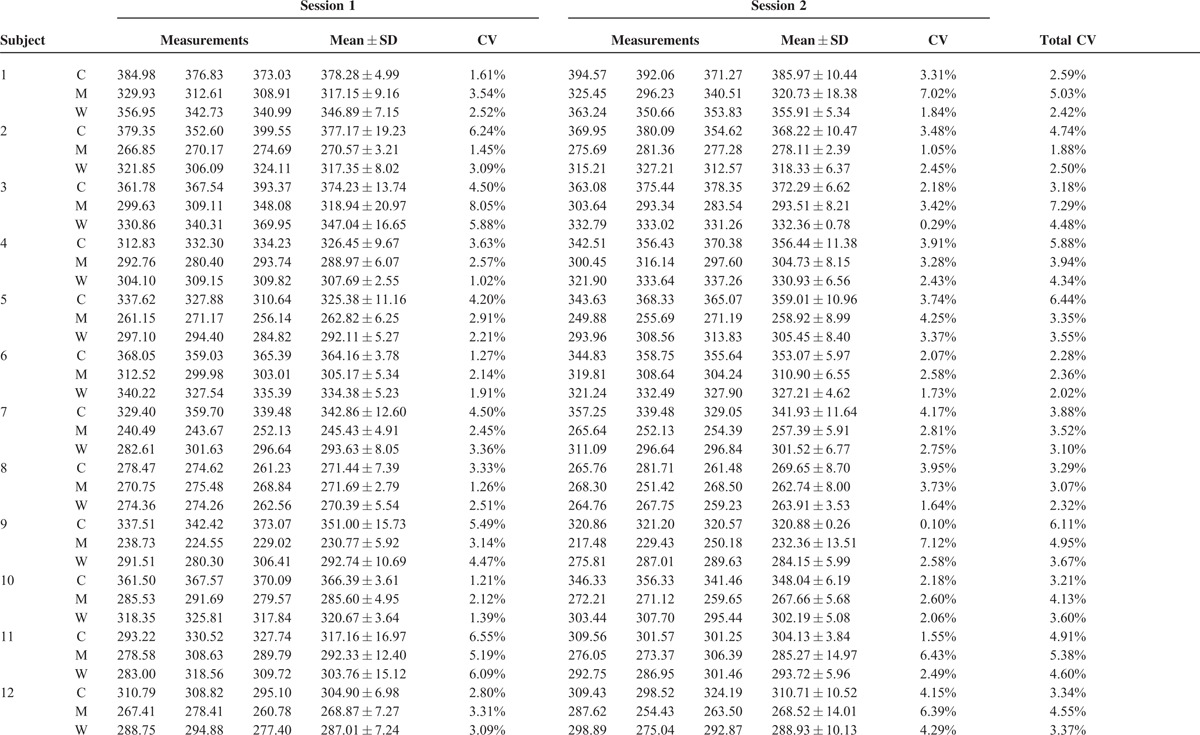
Results of Arterial Spin Labeling Kidney Perfusion Measurements at 1.5 Tesla MRI

**TABLE 1 (Continued):**
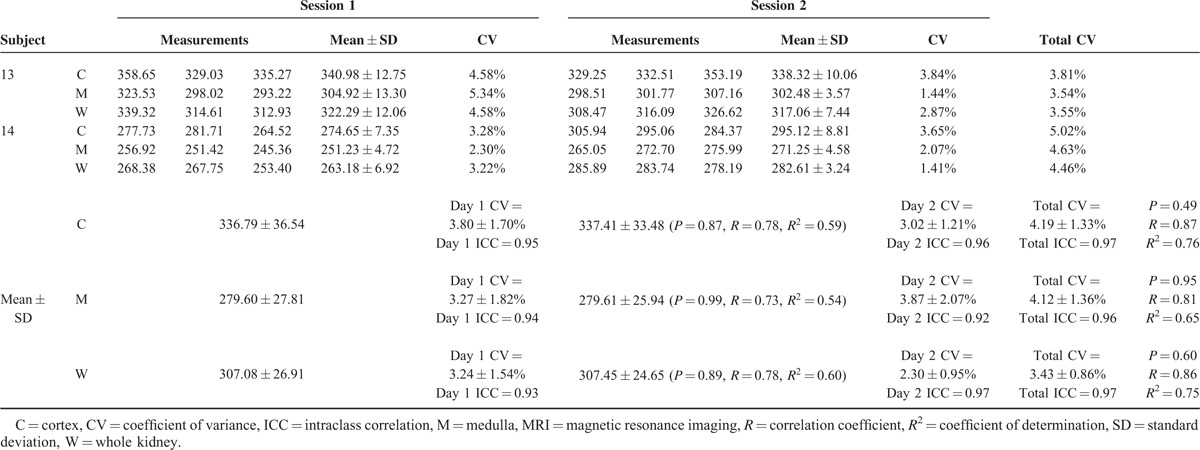
Results of Arterial Spin Labeling Kidney Perfusion Measurements at 1.5 Tesla MRI

## DISCUSSION

The aim of this study was to evaluate the reproducibility of noninvasive ASL kidney perfusion measurements combined with semiautomatic segmentation of the renal cortex and medulla, which allowed the differential quantification of cortical and medullary perfusion in healthy volunteers at 1.5 Tesla MRI. The proposed method revealed high correlation and low variance between 6 repetitive measurements of cortical, medullary, and whole kidney perfusion.

One present constraint is the time consumption and operator dependence required for ASL image analysis. The previously reported mean image analysis time takes approximately 30 minutes per kidney.^29^ Therefore, we propose that ASL, in combination with semiautomatic segmentation of the renal cortex and medulla, allows the differential quantification of cortical and medullary perfusion, which is capable of approximating perfusion in different compartments of the kidney. The applied postprocessing technique is based on a signal intensity threshold facilitating cortical and medullary segmentation. The obtained perfusion values may reveal differences in absolute and relative perfusion in patients with kidney disease. Semiautomatic segmentation presumably generates more reliable, less operator-dependent, and hence more reproducible data. Good reproducibility was demonstrated in the perfusion measurements made at 1.5 Tesla MRI, with within coefficient of variations calculated as 4.2% for cortical perfusion, 4.1% for medullary perfusion, and 3.4% for whole kidney perfusion. These findings are similar to measures of reproducibility found in other studies at 1.5 Tesla.^26,33^ Good reproducibility of ASL was also shown for 3 Tesla.^34,35^ Our study provides further evidence for the reproducibility of ASL at 1.5 Tesla. To ensure uniformity of renal function and minimal variation in scan conditions in our cohort, subjects underwent biochemical screening of blood and urine, as well as physical assessment, to confirm normal kidney function prior to imaging. Furthermore, participants were assessed at a fixed time of day.

Currently, the PAH clearance method is the gold standard to assess renal plasma flow in humans from which the renal blood flow can be determined by a scaling factor dependent on the hematocrit. This technique is time-consuming, with the protocol lasting up to 2 hours.^36^ It also requires intravenous cannulation and involves the risk of complications, such as allergic reactions. It is known that due to the incomplete renal excretion of PAH, the technique underestimates plasma flow by approximately 10%–20% and to a greater extent when plasma flow is less than 300 mL/min.^37^ These limitations support ASL for the assessment of renal perfusion in clinical studies. Although the MRI examination time strongly depends on the applied sequence and scanning protocol, the duration is roughly 8 minutes (which enables 8 repetitive scans), which therefore enables dynamic renal perfusion measurements during therapeutic intervention. This method provides a distinct advantage over PAH and gadolinium contrast-enhanced MRI, which are not repeatable within such a short time frame. The relatively short scan time and the noninvasive character of ASL are also of benefit during recruitment and examination in the context of clinical studies.

Recently published data using ASL to measure renal perfusion in subjects with normal renal function vary widely, with values ranging from 197 to 329 mL/min/100 g.^28,34^ This disparity may represent physiological or population differences. There are other factors that may also affect the ASL method and potentially contribute to these differences. Varying ASL protocols have been employed, differing in breathing technique, field strength, and motion correction, using both single and multislice approaches.^38^ The lack of standardization is a constraint regarding the practice of ASL in clinical routine. ASL appeared to be suitable for quantitative kidney perfusion measurements in patients with renal artery stenosis, as ASL data were related with renal artery stenosis grade and single photon emission CT (SPECT) perfusion values in a cohort of 12 patients with renal artery stenosis, as well as 6 patients with arterial hypertension without renal artery stenosis.^17^ In a recent study, a qualitative agreement between ASL and scintigraphy with respect to kidney perfusion measurements was found.^21^ Another study demonstrated a significant increase in renal perfusion quantified with ASL, using the renin inhibitor aliskiren.^39^ The same group has shown that renal denervation performed in patients with drug-resistant hypertension does not appear to impact renal perfusion, again assessed with ASL.^40^ These data suggest that ASL is a promising method for the assessment of renal hemodynamic changes during therapeutic interventions, without the need for more time-consuming methods, the administration of exogenous contrast agents or the use or ionizing radiation. ASL may potentially provide substantial insights into the pathophysiology of a number of conditions in which renal perfusion is affected, including acute kidney injury, heart failure, and renal arterial disease.^41–43^ Kidney perfusion is also of interest in the context of chronic kidney disease, including status after kidney transplantation.^33^ Furthermore, ASL may add important information during the differentiation of histological subtypes of renal masses.^44^ Rossi et al^45^ recently reported that a moderate renal dysfunction leads to a significant change in the distribution of cortical perfusion and to a reduced cortical and medullary perfusion. Previous studies demonstrated that cortical renal perfusion is 3 to 4 times higher compared to medullary perfusion.^16,17^ Interestingly, our data show a distinctively smaller difference. One explanation for the high perfusion values of the medulla may be attributable to the segmentation since we focused on the correct segmentation of the cortex. Not including parts of the medulla in the segmentation of the cortex was of particular importance. Therefore, the segmentation of the medulla may contain parts of the cortex what explains higher than expected medullary perfusion values. Additionally, the medulla is not as uniform in its makeup as the cortex. The section defined here as the medulla is a band that contains pyramids as well as interlobar veins and arteries.

It should be acknowledged that MRI-based examinations are limited by the relatively high costs, the partly limited access to scanners (e.g., compared to ultrasound), some contraindications (e.g., subjects with some types of metal implants or pacemakers), and the potentially reduced convenience and tolerability for patients (e.g., claustrophobia). In addition to the numerous ASL sequences, various acquisition strategies are applied to minimize the error caused by respiratory motion. Consistent with our approach, other studies have employed breath-holding techniques that minimize respiratory motion, which can prove difficult for participants to comply with. In our study, all of our healthy volunteers were able to comply with an 18-second breath hold; however, this strategy may not be appropriate for handicapped patients. Alternative strategies include prolonged acquisition during free breathing, respiratory triggering, and parallel imaging methods.^46^ The major advantages of ASL methodology in comparison to other methods to measure kidney perfusion (in particular PAH clearance techniques and renal scintigraphy with 99mTc-MAG3) are its noninvasive nature and quick performance within a few minutes. Moreover, because of the high correlation and low variance of ASL measurements, only small numbers of patients are required in studies to detect clinically meaningful changes. For example, in a parallel design of 2 groups with 12 subjects each, a 10% change in whole kidney perfusion can readily be detected following a pharmacologic intervention compared to the control group. This exemplary sample size calculation underscores the high reliability of ASL kidney perfusion measurements.

Nevertheless, ASL kidney perfusion measurements are currently a research tool. Further research and improvements in hard- and software are necessary before it can be implemented into clinical practice. Additionally, future work is required to standardize renal perfusion measurements using ASL.

## CONCLUSION

Noninvasive ASL kidney perfusion measurements at 1.5 Tesla MRI combined with operator-independent semiautomatic quantification revealed a high correlation and low variance of cortical, medullary, and whole kidney perfusion in healthy subjects. This method potentially facilitates insights into the pathophysiology of a number of conditions in which renal perfusion is affected and is a promising tool for the assessment of renal hemodynamic changes during therapeutic interventions. Since a healthy cohort was investigated, further research needs to be done to show if the low variance and high correlation can be translated to detect clinically meaningful changes.
